# Tanshinone IIA Alleviates the AD Phenotypes in APP and PS1 Transgenic Mice

**DOI:** 10.1155/2016/7631801

**Published:** 2016-05-04

**Authors:** Fengling Li, Guosheng Han, Kexiang Wu

**Affiliations:** The Affiliated Hospital of Weifang Medical University, Weifang, Shandong 261031, China

## Abstract

Therapeutic approach for Alzheimer's disease (AD) is still deficient. To find active compounds from herbal medicine is of interest in the alleviation of AD symptoms. This study aimed to investigate the protective effects of Tanshinone IIA (TIIA) on memory performance and synaptic plasticity in a transgenic AD model at the early phase. 25–100 mg/kg TIIA (intraperitoneal injection,* i.p.*) was administered to the six-month-old APP and PS1 transgenic mice for 30 consecutive days. After treatment, spatial memory, synaptic plasticity, and related mechanisms were investigated. Our result showed that memory impairment in AD mice was mitigated by 50 and 100 mg/kg TIIA treatments. Hippocampal long-term potentiation was impaired in AD model but rescued by 100 mg/kg TIIA treatment. Mechanically, TIIA treatment reduced the accumulations of beta-amyloid 1–42, C-terminal fragments (CTFs), and p-Tau in the AD model. TIIA did not affect basal BDNF but promoted depolarization-induced BDNF synthesis in the AD mice. Taken together, TIIA repairs hippocampal LTP and memory, likely, through facilitating the clearance of AD-related proteins and activating synaptic BDNF synthesis. TIIA might be a candidate drug for AD treatment.

## 1. Introduction

Alzheimer's disease (AD) is an aging-related disease hallmarked by the decline of memory performance and other cognitive abilities. It is considered as the most severe neurodegenerative disease, affecting an estimated 25 million people globally [1]. Effective treatment is still lacking in AD due to the complexity of the disease pathology [2–4]. Impairment of synaptic plasticity and pyramid cell loss are thought to render the disease progression. Recent available drugs for AD treatment are mainly working through their activities in promoting cerebral-vascular blood flow [5]. However, those drugs are either too expensive or unsatisfactory for symptoms alleviation.

At the early phase of AD, synaptic transmission and plasticity are affected without loss of pyramid neurons. Cerebral atrophy or cell death is responsible for the cognitive impairment at the later stage of AD [6]. Early prevention or treatment is relatively effective to interfere with AD. A network of signaling pathways regulate synaptic transmission or plasticity. Protein kinase A (PKA), extracellular signal-regulated kinase (ERK), and brain derived neurotrophic factor- (BDNF-) Tropomyosin receptor kinase B (TrkB) signaling pathways are prominent [7–9]. According to previous publications, blockage or deletion of those important pathways leads to impairment of synaptic plasticity and memory [10, 11].

Chinese herbal medicines are popularized for their multi-target effects and low toxic effects [12, 13]. Among the documented herbal medicines,* Salvia miltiorrhiza* Bunge (Danshen) is beneficial for AD treatment [14–16]. Danshen belongs to the Labiatae family of the plant kingdom. Based on the theory of traditional Chinese medicine (TCM), Danshen has the function of activating blood circulation and removing blood stasis, targeting the “heart,” “pericardium,” and “liver” channels [17, 18]. Clinically, Danshen has been widely used to treat various circulatory disturbance-related diseases for its special pharmacological actions, including vasodilatation, anticoagulation, anti-inflammation, and free radical scavenging [19]. Tanshinone IIA (TIIA) is an active lipophilic component extracted from the root of* Salvia miltiorrhiza* Bunge and possesses pharmacological activities of anti-inflammatory, antioxidative, and cytotoxic activity as well as inducing apoptosis. TIIA displayed neuroprotective effects on *β*-amyloid-induced toxicity in rat cortical neurons [20]. Tanshinone, especially, influences the levels of nitric oxide synthase and acetylcholinesterase in the brain of AD rats [21]. However, the effects of TIIA on synaptic plasticity in AD model and related mechanisms are not reported. In this study, an AD transgenic mice model (APPswe, PSEN1dE9) was used to investigate the effects of TIIA on memory and its mechanisms.

## 2. Materials and Methods

### 2.1. Animals

APP/PS1 mice (B6C3-Tg) were purchased from Jackson Lab and bred in our own colony in Weifang Medical University. The offspring was genotyped by primers for APP and PS1. APP/PS1 mice have accelerated the AD phenotype characterized by increased A*β* deposits and behavioral deficits as young as 13–16 weeks [22]. The 6-month-old male mice were used in the experiments and housed in a 12 h light/dark cycle with food and water* ad libitum*. Age- and sex-matched C57BL/6J mice (Animal Center of Chinese Academy of Sciences, China) were used as Controls. All the experimental procedures were approved by Weifang Medical University.

### 2.2. Tanshinone IIA Treatment

Tanshinone IIA (Santa Cruz, USA) by intraperitoneal injection (*i.p.*) (25 mg/kg, 50 mg/kg, and 100 mg/kg) was chronically administered to the mice once each day for 30 consecutive days. This dose range was chosen based on previous publications [23, 24]. TIIA (16 mg/kg) readily penetrated the blood brain barrier reaching a peak concentration of 0.41 nmol/g brain wet weight 60 minutes after intraperitoneal injection [25]. TIIA was dissolved in DMSO and diluted in saline. Control and AD mice received the similar volume of vehicle (DMSO diluted in saline). Diet, drink, and body weight were monitored during the drug administration. 30 days after administration, behavioral, electrophysiological, and biochemical experiments were carried out.

### 2.3. Electrophysiological Experiments

Mice were anesthetized by ether and decapitated. The brain was isolated quickly on ice. After that, acute hippocampal slices (400 *μ*m) were prepared in cutting solution (124 NaCl, 26 NaHCO_3_, 10 D-glucose, 3 KCl, 1.25 KH_2_PO_4_, 5 MgSO_4_, and 3.4 CaCl_2_). The slices were then transferred to an interface recording chamber and exposed to a warm, humidified atmosphere of 95% O_2_/5% CO_2_ and continuously perfused with oxygenated and preheated (32 ± 0.5°C) artificial cerebrospinal fluid (aCSF) (in mM) [110 NaCl, 5 KCl, 2.5 CaCl_2_, 1.5 MgSO_4_, 1.24 KH_2_PO_4_, 10 D-glucose, and 27.4 NaHCO_3_] with a speed of 1.5 mL/min. The field EPSP was elicited by stimulating Schaffer collateral pathway with twisted nichrome wires. The input-output and paired-pulse facilitation at 30 ms, 50 ms, and 100 ms intervals were tested. Long-term potentiation was triggered by theta-burst stimulation (TBS, 10 bursts of four pulses at 100 Hz delivered at 5 Hz interval).

### 2.4. ELISA

Beta-amyloid 1–42 was quantified by ELISA method. The hippocampus from different groups were homogenized in homogenization buffer (5 M guanidine HCl/50 mM Tris–HCl) and centrifuged. Protein concentrations of supernatants were determined using a BCA kit (Thermo Fisher Scientific, USA). Supernatant fractions were analyzed by beta-amyloid 1–42 ELISA kit (KHB3441, Invitrogen, Carlsbad, CA, USA) according to the manufacturer's protocol. Absorbance was determined for each well at 450 nm using a microplate reader (Thermo Scientific, USA).

### 2.5. Real-Time PCR

Total RNA was extracted from hippocampus using Trizol reagent (Invitrogen, Carlsbad, USA). Reverse transcription was carried out using Moloney murine leukemia virus reverse transcriptase (Promega, Madison, USA). Real-time PCR was performed for the quantification of APP in hippocampus with a quantitative thermal cycler (Mastercycler ep realplex, Eppendorf, Germany). Relative expression values were calculated as the ratio of target cDNA to *β*-actin. The primers used in real-time PCR were listed as follows:

 APP: sense primer 5′-TGCTGGCAGAACCCCAGATCG-3′; antisense primer 5′-TTCTGGATGGTCACTGGCTGG-3′;
 
*β*-actin: sense primer 5-ATGAGGTAGTCTGTCAGGT-3; antisense primer 5-ATGGATGACGATATCGCT-3.

### 2.6. Biochemical Experiments

Acute hippocampal slices were incubated with normal aCSF. One hour after recovery, slices from different groups were depolarized by KCl (90 mM, 3 min). The concentration of NaCl in KCl-aCSF was reduced making the composition of KCl-aCSF as follows: 37.5 mM NaCl, 90 mM KCl, 1.25 mM NaH_2_PO_4_, 25 mM NaHCO_3_, 2 mM CaCl_2_, 1 mM MgCl_2_, and 25 mM glucose. One hour after depolarization, the slices were collected in dry ice and kept at −80°C until use.

Hippocampus homogenates were obtained and lysed. Protein concentrations were measured using BCA protein assay kit (Thermo, US). Equivalent amounts of proteins were processed for SDS-PAGE and western blot. The primary antibodies used were BDNF (1 : 1000, Millipore), Actin (1 : 10000, Millipore), p-Tau (1 : 3000, Cell Signaling), and CTFs (1 : 3000, Cell Signaling).

### 2.7. Morris Water Maze

Morris water maze was conducted in a circular pool with a 150 cm diameter filled with a depth of 22 cm water (25 ± 2°C). A circular Plexiglass platform (8 cm diameter) was placed 2 cm below the water level. Distinctive visual cues were set. A video camera was positioned above the water maze. The swim was tracked, digitized, and stored for later behavioral analysis using EthoVision 3.1 (Noldus). The water maze was divided into four logical quadrants that served as starting positions for the rats.

The spatial learning task consisted of a 5-day acquisition using the hidden platform. This was followed by a probe trial on the sixth day without the platform. The platform was fixed in the middle of the west quadrant, 45 cm from the maze wall. During the first 5 days, four swim trials were given per day, in which each animal was released from a different quadrant in each trial. This was done in a pseudorandom manner and the start quadrant used was varied across the sessions. A maximum of 60 s was allowed for each trial. If the rat did not find the platform within 60 s, it was guided to the platform and allowed to remain there for 10 s. The latency to escape onto the platform was recorded.

### 2.8. Statistical Analyses

Data are presented as means ± SEM. All the statistical analyses were performed by one-way ANOVA with GraphPad Prism 6.0. Bonferroni correction for post hoc *t*-test was performed to compare the differences between groups. *P* value less than 0.05 was considered statistically significant.

## 3. Results

### 3.1. TIIA Ameliorates Spatial Memory in AD Mice

**Figure 1:**
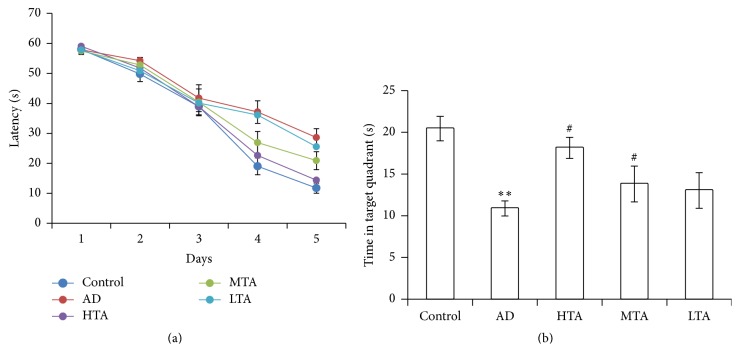
Tanshinone IIA improved the spatial memory in AD mice. (a) Changes of escape latency to reach the hidden platform during the 5 d acquisition trails; (b) the time spent in the target quadrant 24 h after the last acquisition trial. The data were presented as Mean and SEM. In each group, there were 10 animals. High dose (100 mg/kg), medium dose (50 mg/kg), and low dose of Tanshinone IIA (25 mg/kg) were administered to the mice for 30 consecutive days, respectively. ^*∗∗*^ *P* < 0.01 compared with Control and ^#^ *P* < 0.05 compared with AD.

The diet, drink, and weight were not affected during TIIA treatment. After a period of 30-day treatment, spatial memory was evaluated. Within the five-day training section, 100 mg/kg and 50 mg/kg TIIA remarkably decreased the latency to find the platform (Figure 1(a)). On the sixth day, the probe was removed. As shown in Figure 1(b), the time spent in the targeted quadrant was significantly decreased in AD mice when compared to Control mice (*P* < 0.05). By contrast, TIIA treatment significantly reversed the deficit (*P* < 0.05). These data suggested that TIIA mitigated the impaired spatial memory in the AD model.

### 3.2. TIIA Reverses the Deficit of Long-Term Potentiation in AD Mice

**Figure 2:**
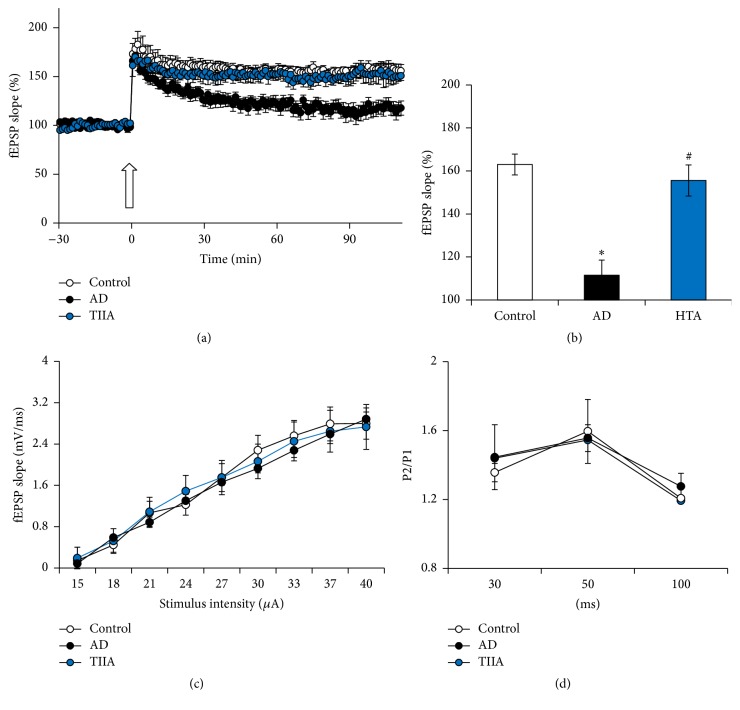
Tanshinone IIA reversed the impairment of long-term potentiation in AD mice. (a) Impairment of TBS-induced LTP in AD model was improved by Tanshinone IIA administration. (b) LTP level at the 90th min after TBS. (c) Input-output was not affected by Tanshinone IIA application. (d) Paired-pulse facilitation was not affected by Tanshinone IIA application. The data were presented as Mean and SEM. 5–10 slices from five animals were included in each group. ^*∗*^ *P* < 0.05 compared with WT and ^#^ *P* < 0.05 compared with AD.

As evidenced by previous study, hippocampal LTP was also impaired at the early phase of the AD model [6]. To confirm the effect of TIIA on hippocampal synaptic transmission and plasticity, fEPSP at Schaffer collateral-CA1 synapses was measured. TBS-induced LTP was impaired in the slice obtained from the AD mice (*P* < 0.05 versus Control) (Figures 2(a) and 2(b)). However, after TIIA treatment, TBS-LTP was mitigated (*P* < 0.05 versus AD model). As shown in Figures 2(c) and 2(d), TIIA did not affect the basal synaptic transmission, including input-output and paired-pulse facilitation. These results suggest that TIIA could ameliorate the synaptic deficit at the early phase of AD.

### 3.3. TIIA Attenuates AD-Related Protein Expression

**Figure 3:**
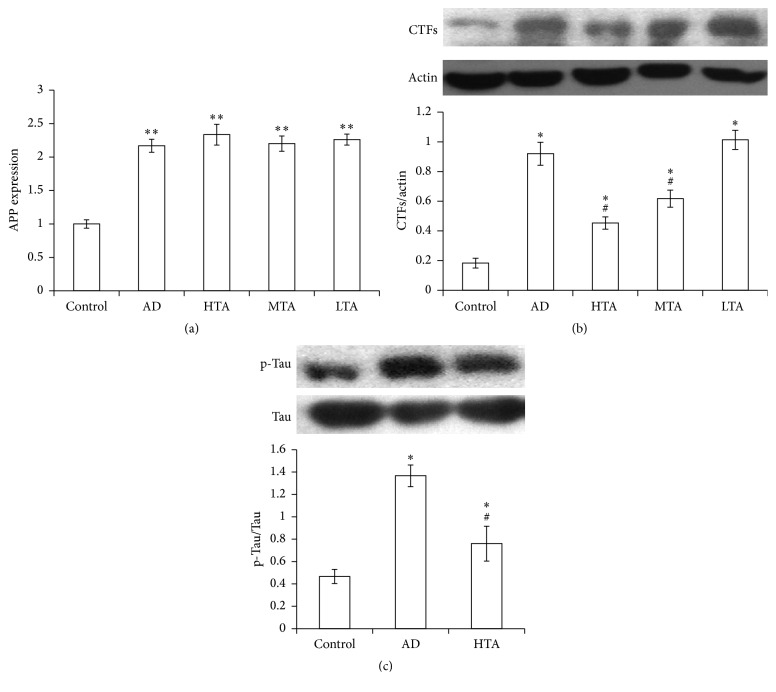
Tanshinone IIA downregulated CTFs and p-Tau expression. (a) APP expression in mRNA level was increased in AD mice. (b) CTFs expression was increased in AD mice, while it was decreased by Tanshinone IIA treatment. Representative blots of CTFs and Actin are inserted above. (c) p-Tau expression was increased in AD mice, while it was decreased by Tanshinone IIA treatment. Representative blots of p-Tau and total Tau are inserted above. The data were presented as Mean and SEM from five animals in each group. ^*∗*^ *P* < 0.05, ^*∗∗*^ *P* < 0.01 compared with Control and ^#^ *P* < 0.05 compared with AD.

**Figure 4:**
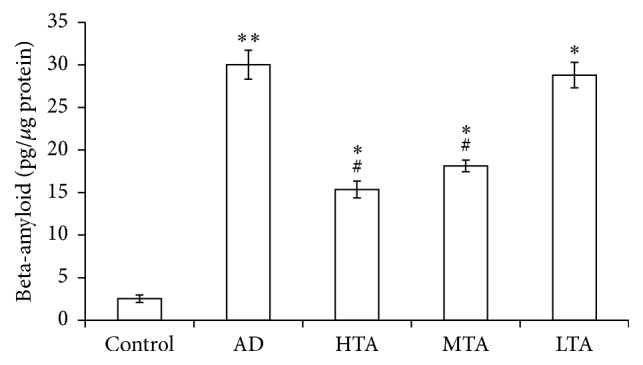
Tanshinone IIA downregulated beta-amyloid 1–42 level in AD model. The data were presented as Mean and SEM from five animals in each group. ^*∗∗*^ *P* < 0.01, ^*∗*^ *P* < 0.05 compared with Control and ^#^ *P* < 0.05 compared with AD.

We detected APP expression in hippocampus by RT-PCR. Compared to wild type mice, APP expression in mRNA level was significantly elevated in AD mice (*P* < 0.05 versus Control). By contrast, TIIA did not alter the APP expression (Figure 3(a)). We also detected CTFs expression. In the model mice, CTFs expression was significantly increased (*P* < 0.05 versus Control); however, it was decreased by TIIA treatment (*P* < 0.05 versus AD model) (Figure 3(c)). As compared with wild type mice, p-Tau expression increased significantly in model mice (*P* < 0.05 versus Control). By contrast, TIIA treatment also reduced this protein level (*P* < 0.05 versus AD model) (Figure 3(c)). In addition, TIIA treatment apparently reduced beta-amyloid 1–42 level in AD mice (*P* < 0.05 versus Control) (Figure 4). These results suggested that TIIA treatment mitigated the accumulation of AD-related protein expression in AD mice.

### 3.4. TIIA Improves Synaptic Activation-Induced BDNF Synthesis in AD Mice

**Figure 5:**
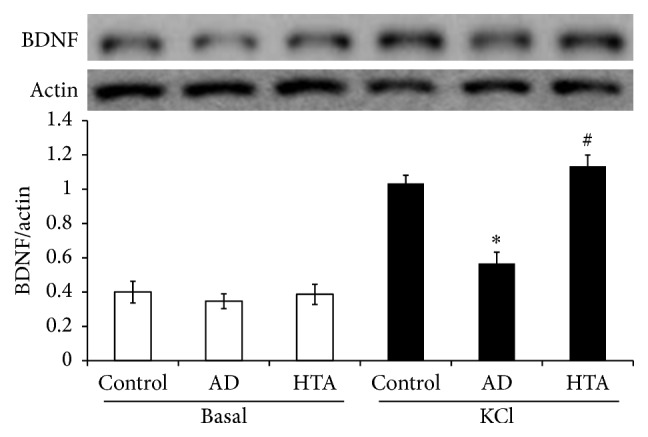
Tanshinone IIA activates KCl-induced BDNF synthesis. The data were presented as Mean and SEM from five animals in each group. ^*∗*^ *P* < 0.05 compared with Control and ^#^ *P* < 0.05 compared with AD.

We also detected synaptic related protein expression. BDNF expression was not remarkably altered in basal level of this AD model. Moreover, TIIA treatment did not promote the protein expression. We analyzed the synaptic activation-induced BDNF synthesis. High concentration of KCl was applied to trigger BDNF synthesis [26]. As shown in Figure 5, BDNF level was significantly elevated after KCl incubation in Control mice. By contrast, KCl-induced BDNF synthesis was blocked in the AD mice. After TIIA treatment, KCl-induced BDNF synthesis was significantly improved (*P* < 0.05 versus AD model).

## 4. Discussion

We demonstrated in this study that TIIA treatment improved the memory and hippocampal LTP in AD model. AD-related protein expressions were effectively reduced and synaptic activation-induced BDNF was improved after TIIA treatment.

Danshen has the pharmacological activities to improve memory in different disease models. Compound Danshen ameliorated cognitive deficit in A*β*25–35 peptide-induced rat model of Alzheimer's disease [14, 27].* Salvia miltiorrhiza* injection protects against memory impairments in streptozotocin-induced diabetic rats [28, 29]. In addition, the formula HX106N with Danshen as the main component was also reported to ameliorate memory deficit in AD model [30]. These consistent reports revealed the potential activity of Danshen in the treatment of AD. As the major active compound in Danshen, TIIA might be one of the active substances exerting the memory improving function. Lam et al. reported that TIIA was detected in blood within 5 minutes after intraperitoneal injection and also detected in the brain 5 minutes after injection, showing that the drug was able to penetrate the blood brain barrier. They also revealed that TIIA (16 mg/kg) readily penetrated the blood brain barrier reaching a peak concentration of 0.41 nmol/g brain wet weight 60 minutes after intraperitoneal injection [25]. In our study, we chose a dose range from 25 to 100 mg/kg and revealed that 50–100 mg/kg of TIIA could effectively improve the memory in AD mice.

The “amyloid hypothesis” indicates that amyloid clearance is an effective strategy for the treatment or prevention of AD. Inhibitors of secretase prohibit the overall A*β* production. However, preclinical study indicated that semagacestat, an inhibitor of secretase, did not show any significant slowing of AD phenotypes [31]. In APP/PS1 transgenic mice, APP was overexpressed, leading to increase of amyloid 1–42 accumulation in hippocampus. In addition, the AD protein p-Tau and CTFs were also enhanced at 6-month-old AD model. In TIIA-treated mice, APP expression was not affected. However, p-Tau, CTFs, and amyloid 1–42 accumulation in hippocampus were significantly reduced. In previous reports, estrogen-mediated PI3K/Akt signaling promotes *α*-secretase cleavage of APP and inhibits the production of A*β* [32, 33]. As a new identified member of the phytoestrogen family [34, 35], TIIA possibly mediates PI3K/AKT signaling pathway to cleavage of secretase and contributes to the decrease of A*β* production. Besides the direct effects on A*β* production, TIIA also likely affects the degradation of A*β*. The elevation of activity of ubiquitin proteasome system by parkin overexpression facilitates the A*β*-related protein clearance [6]. It is possible that TIIA treatment increased the proteasome activity to facilitate A*β* clearance [36].

At the early phase of AD, impairment of synaptic plasticity rather than cell loss contributes to the memory deficit. Although it is difficult to ascertain the direct AD stage displayed by the transgenic AD mice (6-7 months), it is conceivable that the mice were still at the early phase of AD progression, as we did not observe cell loss or impairment of input-output of the basal synaptic transmission. By contrast, hippocampal LTP was impaired in the AD mice. TIIA treatment reversed the impairment LTP but did not affect basal synaptic transmission. Amyloid plague is supposed to be the detrimental toxin contributing to the impairment of hippocampal synaptic plasticity and hippocampal cell death [37–39].

A lot of signaling pathways are involved in the modulation of hippocampal synaptic plasticity [7]. BDNF-TrkB pathway is especially important for the formation of memory and LTP consolidation [10]. BDNF-TrkB was impaired to affect memory in many disease models [8, 40]. Moreover, not only the basal BDNF level but also synaptic activation-induced BDNF synthesis was reduced [41]. In our study, the basal BDNF level was not affected at the early phase of AD mice. However, KCl-induced BDNF synthesis was impaired in the AD model. After TIIA treatment, the abnormality was rescued. In combination with the effects of TIIA on AD-related protein expression, TIIA possibly interferes with the protein accumulation to reverse the synaptic activation-induced BDNF synthesis.

## 5. Conclusion

In this study, we provided data revealing the memory improving effect after TIIA treatment in a transgenic AD model. The clearance of AD-related protein and activation of BDNF-TrkB pathway might possibly contribute to the effect of TIIA on hippocampal LTP and memory. These results implicated that TIIA is a potential memory improver in AD model.
